# Histopathological discrepancy and variation of surgical management in mucinous ovarian cystadenoma and pseudomyxoma peritonei

**DOI:** 10.1016/j.ijscr.2022.107141

**Published:** 2022-05-03

**Authors:** Gatot Purwoto, Tricia Dewi Anggraeni, Primariadewi Rustamadji, Ilham Utama Surya, Kelli Julianti, Nathaniel Herlambang

**Affiliations:** a Faculty of Medicine University Indonesia, Department of Obstetrics and Gynecology, Dr. Cipto Mangunkusumo Hospital, Jakarta, Indonesia; b Faculty of Medicine University Indonesia, Department of Anatomic Pathology, Dr. Cipto Mangunkusumo Hospital, Jakarta, Indonesia

**Keywords:** Pseudomyxoma peritonei, Mucinous cystadenoma, Cytoreductive surgery

## Abstract

**Introduction and importance:**

Mucinous cystadenoma occurs in 10–15% of all ovarian tumors. Diagnosis and treatment should be decided precisely as it has a chance to develop into pseudomyxoma peritonei (PMP). Management of PMP might be challenging especially when repeated surgery is needed.

**Case presentation:**

The first case, a 22-year-old lady with recurrent stomach enlargement for seven months. She had history of laparotomy surgery due to an ovarian tumor. Whole abdomen contrast CT scan showed a large cyst with mucinous fluid. We decided to do re-laparotomy and found a left ovarian cyst. Histological examination results confirm ovarian mucinous cystadenoma. The second case was, 55-year-old woman, with abdominal enlargement for six months. She had a history of laparotomy and chemotherapy due to pseudomyxoma peritonei. Post chemotherapy MRI showed persistent pseudomyxoma and two multilocular cysts from both adnexa. Debulking laparotomy was then conducted. We obtained 8 L of mucinous pseudomyxoma along with mucinous cyst from both ovaries. The final diagnosis concluded as a pseudomyxoma and we decide to close the follow-up of the patient.

**Clinical discussion:**

Pseudomyxoma is caused by the production of mucin originating from intra-abdominal organs. Open surgery should be prioritized when the mucinous cystadenoma is detected to do a complete peritoneum evaluation and avoid perioperatively ruptured mucinous neoplasm. Pseudomyxoma often needed repeated surgical treatment and may exhibit different surgical findings and different pathologies.

**Conclusion:**

Repeated surgery is logical and still no need for adjuvant chemotherapy in both cases. Accurate and precise diagnosis should be prioritized in order to prevent repeated surgery.

## Highlights

• Accurate and precise diagnosis seems need to be prioritized in order to prevent repeated surgery.
• Histopathological discrepancies need the expertise of the pathologists who specialize in gynecologic oncology.
• Repeated surgery is logical and generally no need for adjuvant chemotherapy.

## 1 Introduction

Among cases of ovarian tumor, mucinous cystadenoma occurs in 10–15% of all [1]. Cystadenoma tumors consist of mucin-producing epithelial cells [2], [3]. Mucinous cystadenoma is considered a benign tumor. The ruptured tumor can lead to pseudomyxoma peritonei (PMP) formation [1].

PMP is a rare condition with an estimated incidence of approximately one per million per year and characterized by the production of vast amounts of mucinous fluid [4], [5]. Peritoneal fluid and gravity help these cells move intra-abdominally [6], [7]. Due to the disease's variable clinical appearance, diagnosing PMP can be confusing [8].

Management of PMP might be challenging, because it may involve repeated surgery and chemotherapy. Diagnosing mucinous cystadenoma should be decided precisely, regarding the treatment as a chance of becoming PMP [9]. We will elaborate on two cases with some differences and similarities of variation in management.

## 2 Case description

### 2.1 Case I

A 22-year-old lady came with recurrent enlargement of the stomach for seven months. She had history of laparotomy surgery in 2015 due to an ovarian tumor. The histopathological result showed immature teratoma of the right ovary and endometriosis of the left ovary. In 2019, she had recurrent stomach enlargement and underwent surgery (right salpingo-oophorectomy, omentectomy, and appendectomy). Histopathological results of this surgery showed right ovarian mucinous cystadenoma, inflammation of the omentum and a normal appendix.

**Fig. 1:**
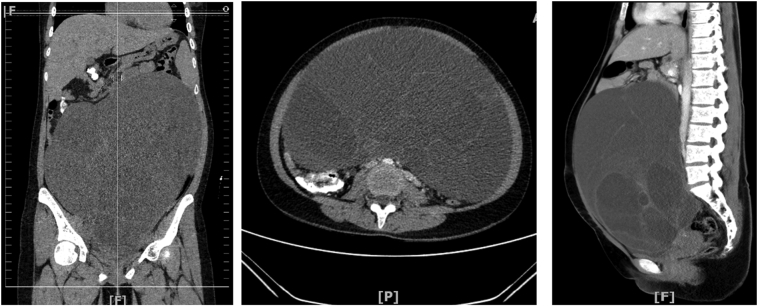
CT whole abdomen with contrast showed a large cystic multilocular septated lesion with mucinous fluid.

Again in 2021, she came to our hospital with a third recurrent. The abdominal mass limited mobilization sized 30 cm in diameter. Laboratory findings found CEA 1.0 ng/ml, Ca 19–9934.6 U/ml, Ca- 125 24.9 U/ml. Whole abdomen contrast Computed Tomography (CT) scan showed a large cystic multilocular septated lesion with mucinous fluid and few solid components in the pelvic cavity and upper abdomen (Fig. 1). We decided to do re-laparotomy for conservative surgery, supported with a frozen section procedure.

**Fig. 2:**
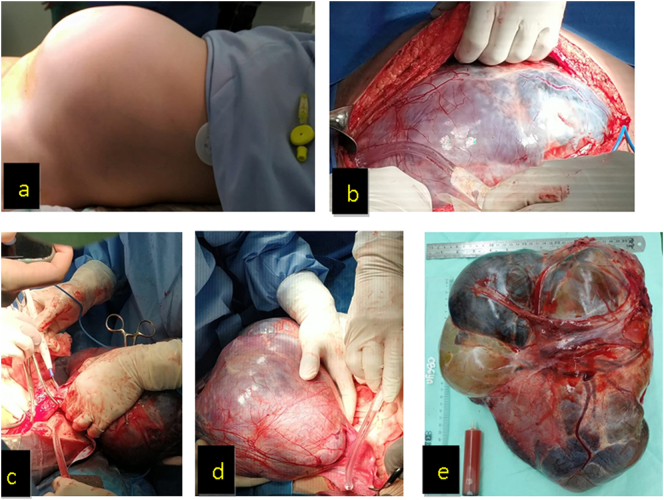
(a–e). Ovarian cystadenoma originating from left ovary with 40 cm diameter.

Intra-operative findings were 25 cm^3^ ascites, left ovarian cyst diameter of 40 cm. Fibrin adhesion with a rectosigmoid colon. Uterine was normal-sized and there was no more right adnexa (history of prior surgery). Left salpingo-oophorectomy was performed and the frozen section result was benign mucinous cystadenoma (Fig. 2). After confirming with paraffin block, the histopathological result was mucinous cystadenoma with proliferative borderline focals. After the final surgery findings, cytology, histopathology result, and data from prior surgeries lead to the final diagnosis of ovarian mucinous cystadenoma.

### 2.2 Case II

**Fig. 3:**
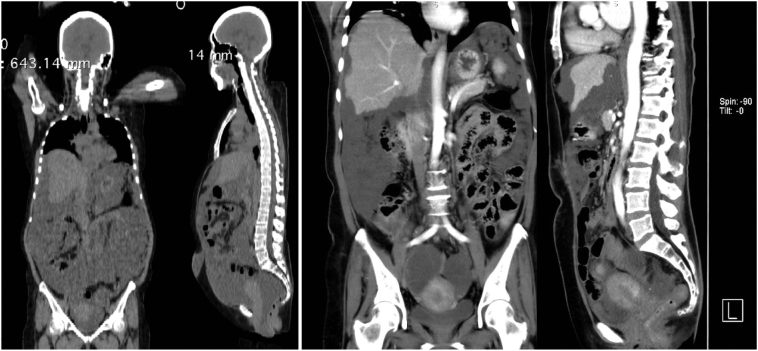
CT-Scan whole abdomen: A. Pre surgery (July 2020) showing ascites in the abdominopelvic cavity, B. post surgery (October 2020) showing fluid accumulation and multilocular cystic mass in the abdominopelvic cavity.

Mrs, 55-year-old, P3A0, complained about abdominal enlargement for six months. She was treated by Internist. Abdominal ultrasound results showed massive intra-abdominal ascites, followed by ascites aspiration. The result was negative for malignancy and negative for PCR-TB. She was then referred to an oncology-surgeon and performed exploratory laparotomy. The surgical procedure was omentectomy and biopsy done. Specimen pathology result was peritoneal pseudomyxoma. Then the patient was referred to Internist in Cipto Mangunkusumo hospital. A whole abdomen CT-Scan results: fluid accumulation fulfill the entire intraperitoneal cavity. A well-defined multilocular cystic mass was found, sized of 6.2 × 5.6 × 6.7 cm (right) and 5.4 × 5.1 × 5.8 cm (left) (Fig. 3).

The patient was planned for HIPEC procedure, but due to not being feasible to manage, the patient was decided to change treated with FOLFOX regimen (leucovorin calcium, fluorouracil, and oxaliplatin) and finally receive 5-fluorouracil (FUFA) and leucovorin according to availability of the national health insurance.

**Fig. 4:**
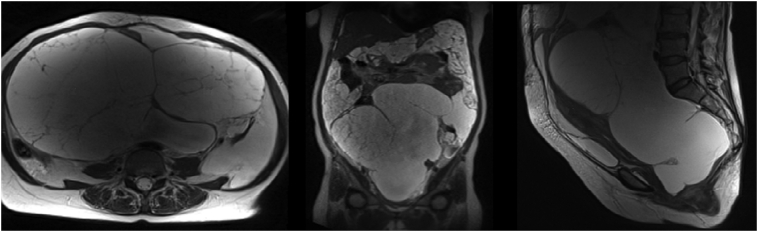
MRI whole abdomen showed accumulated fluid filling the entire intraperitoneal cavity and two multilocular cysts after six courses of chemotherapy.

After completing six courses chemotherapy, MRI was performed, and the results showed that accumulated fluid appeared heterogeneous after contrast, filling the entire intraperitoneal cavity from the upper to lower abdomen. Two multilocular cysts filled the pelvic cavity to the abdominal cavity measures approximately 14.6 × 21.8 × 30.5 cm (right) and 9.5 × 11.0 × 12.9 cm (left) (Fig. 4).

The patient was consulted to Onco-Gynecology regarding an ovarian mass. Examination at the gynecological oncology distended abdomen with an abdominal girth of 100 cm. Inspeculo examination found a posterior vaginal bulging. Rectovaginal examination showed the mass filled the abdominal-pelvic cavity to fill the Douglas cavity. Laboratory examination showed CEA 117 ng/ml, CA-125 46 U/ml, and CA19-9 311 U/ml.

**Fig. 5:**
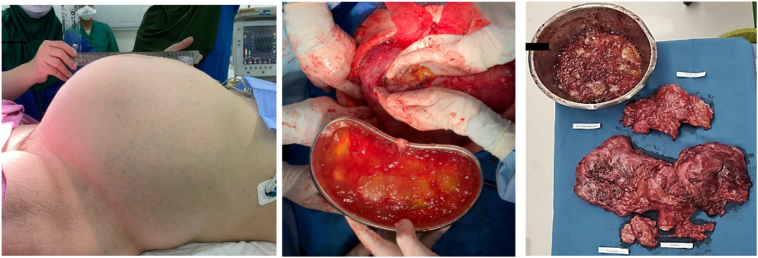
A. Enlarged abdomen with fluid accumulation and adnexal mass. B. Eight liters of jelly fluid. C. Macroscopic surgical specimens after surgery.

The patient was planned for a debulking laparotomy and a total hysterectomy. On exploration, there was 8 L of mucinous pseudomyxoma obtained from the abdominal and pelvic cavities. Both mucinous ovarian cysts diameter were 15 cm with massive spreading mucinous implant until mesocolon, mesoilleum, subhepatic and subdiaphragmatic area. Surgical procedures were total abdominal hysterectomy, bilateral salpingo-oophorectomy, omentectomy, partial (pararectal) peritonectomy, but not reach subdiaphragma stripping peritonectomy due to risk of diaphragma perforation. The abdominal cavity was then washed with 5% dextrose and 4 L of distilled water. A 32F drain catheter was installed for the preparation of intraperitoneal chemotherapy (Fig. 5).

**Fig. 6:**
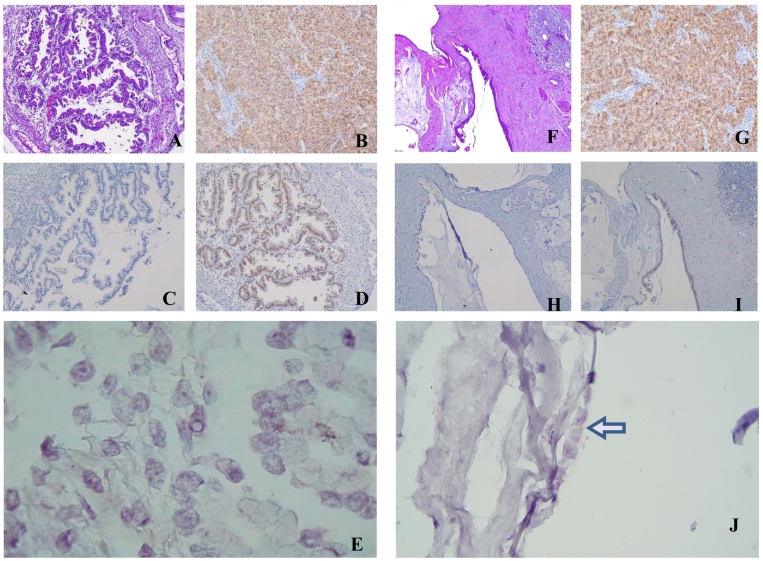
Immunohistochemistry labeling results (obj ×100). A. Mucinous cystadenoma with borderline focus HE staining. B. Positive control: +3 score HER2. C. Negative control HER2. D. +2 result HER2: equifocal weak-to-moderate complete membrane staining. E. Immunopathology CISH ratio HER2/CEP17: 2.27 and an average HER2 signals/cell: 5.45 (HER2 positive). F. Pseudomyxoma Hematoxylin-Eosin (HE) staining. G. Positive control: +3 score HER2. H. Negative control HER2. I. Negative result of HER2. J. Immunopathology CISH Ratio HER2/Cen17: 1.3 (negative).

As the previous discussion with internal medical-oncologist, surgical procedure performs pigtail drain catheter for HIPEC preparation procedure later after surgery. Histopathological results of surgical specimens showed mucinous pseudomyxoma of the fluid and the mucinous containing component of the ovarian cystadenoma. The final diagnosis concluded as a pseudomyxoma peritonei and decided no more treatment for HIPEC or chemotherapy and just closed follow-up (Fig. 6).

In these cases, *HER2* expression was found in borderline mucinous cystadenoma, but not found in PMP. It seems that positive *HER2* protein was not detected in normal or benign tumor. Borderline tumors show *HER2* positive staining, but mostly no overexpression [16]. Chromogenic in situ hybridization (CISH) analysis was calculated from 20 tumor cell nucleus with ratio of HER2/CEP17 (cystadenoma) and HER2/Cen17 (pseudomyxoma). HER2 status categorized as HER2 positive when the HER2/CEP17 ratio is ≥2.0 and the average HER2 gene copy number is ≥4.0 per tumor cell. In first case (mucinous cystadenoma), ratio HER2/CEP17 was 2.27 and an average HER2 signals/cell was 5.45, it showed that HER2 positive. Interpretation of ratio HER2/Cen17 were (<1,8) negative, (1.8–2.2) equivocal, and (≥2.2) positive or amplification. CISH result of pseudomyxoma was 1.3 (negative). It indicates no amplification in the tissue.

## 3 Timeline

**Table:** 

Date	Case I	Case II
2015	• February: Laparotomy surgery Histopathological result: immature teratoma of the right ovary and endometriosis of the left ovary	
2019	• April: Repeated laparotomy Histopathological results: right ovarian mucinous cystadenoma	
2020	–	• July: Laparotomy surgery by oncology-surgeon was performed (omentectomy & biopsy). Result: PMP • October: Whole abdomen CT-Scan was performed Six courses chemotherapy with FOLFOX regimen, FUFA and leucovorin
2021	• June: Left salpingo-oophorectomy Histopathological result: benign mucinous cystadenoma	• July: MRI post chemotherapy was performed • November: Debulking laparotomy + total hysterectomy were performed. Histopathological result: mucinous pseudomyxoma + ovarian cystadenoma.

## 4 Discussion

Pseudomyxoma can occur and caused by the production of mucin originating from intra-abdominal organs, including the ovaries, appendix, intestines, or peritoneal wall. First case illustrates that the final pathological result is a mucin cystadenoma that may cause ascites pseudomyxoma, but the ascites obtained do not contain mucin or pseudomyxoma elements to be included in the possibility of an early stage of pseudomyxoma. There was a different histopathology result from the first to the third examination in the first case. It revealed that the pathological examination findings at the first or subsequent surgery might be different because there are indeed differences in the components of the tissue structure in one part of the ovary, or it could be due to the transformation of cells from the ovary. Likewise, pathological examination with frozen section, it is possible that the results will be inaccurate compared to the results of paraffin block pathology. Such a condition was due to the accuracy of the frozen section, which significantly lower for mucinous ovarian tumors than other histological types. The causes of misdiagnosis in frozen section biopsy are mucinous ovarian tumor larger than other histological types, lower number of frozen section slides, experience or expertise of the pathologists who not specialized in gynecology oncology [10]. Open surgery should be prioritized when the mucinous cystadenoma is detected to do a complete peritoneum evaluation to avoid perioperative ruptured of mucinous neoplasm [11].

In the second case, despite having given a chemotherapy regimen on the first surgical biopsy findings that showed pseudomyxoma, it did not respond, even the mucin production was increasing. Further planning by the surgical team will be debulking and preparation for HIPEC chemotherapy, but the findings during the surgery showed that all components of ascites and right and left ovarian cysts contained mucin material and were not malignant. Based on the findings of the latest pathology results were pseudomyxoma, no malignant cells were found, it was decided that the therapy was only close follow-up without chemotherapy.

We only did cytoreductive surgery based on our second case without HIPEC procedure. According to 5-year survival rates study conducted by Jarvinen et al., there was no difference between cytoreductive surgery alone compared to combination cytoreductive surgery and intraperitoneal chemotherapy [12]. Another study showed a similar result when comparing cytoreductive surgery alone with cytoreductive surgery and HIPEC combination, producing indistinguishable survival outcomes. Cytoreductive surgery was undertaken via a long midline incision from xiphisternum to the symphysis pubis and involved peritonectomy or stripping of the peritoneum from up to six regions of the abdomen combined with visceral resections of involved organs. The procedures include omentectomy, appendectomy, and may involve splenectomy, cholecystectomy, and right hemicolectomy [13].

Pseudomyxoma cases often receive repeated surgical treatment and may exhibit different surgical findings and different pathologies. However, in conclusion, both cases showed mucin cysts and pseudomyxoma which did not require chemotherapy [15]. According to Ahmadi et al. study, which involved 430 patients, the comparison overall survival of patients with recurrent PMP showed a better outcome in a group of cytoreductive surgery alone than in a group of watch-and-wait or palliative chemotherapy [14].

## 5 Conclusion

Repeated surgery is logical and still no need for adjuvant chemotherapy in both cases. Accurate and precise diagnosis should be prioritized in order to prevent repeated surgery.

### 5.1 Patient's perspective (first case)

The third time of my stomach enlargement for seven months in 2021. My haid cycles were regular within the four last months. My doctor asks me whenever I had nausea, abdominal pain, and fever, but I felt none of it. I took Whole abdomen contrast Computed Tomography (CT) scan as a doctor's recommendation. And finally, the doctor decided to do re-surgery with informed consent that if my left ovary was not normal or don't have any function anymore, it may be decided to remove it. All of those considerations are based on the pathologist's examination in the middle of surgery. The final diagnosis of ovarian mucinous cystadenoma. And the medical team took a very hard decision to remove my left ovary, because it may always be a possibility of recurrence in the future. My family and I accept this hard decision, but we know that it will the best treatment for my condition.

### 5.2 Patient's perspective (second case)

The first symptom I felt was the enlargement of my stomach. At first, I just thought it was due to normal weight gain. However, as it turned out, my stomach grew rapidly in a relatively short period. After my first surgery in 2020 and chemotherapy, I felt a sense of relief, but soon the feeling of fullness in my stomach returned. In the end, I decided to be referred to a gynecologic oncology specialist. The discovery of 2 lumps from my ovaries and the accumulation of fluid in my abdominal cavity made the symptoms of shortness of breath appear. The 2nd operation was then conducted, and it went well. I felt that my abdominal circumference was much smaller, and the feeling of tightness in the abdominal cavity was reduced. I was then observed for four days post operative and then discharged.

## Patient consent

Written informed consent was obtained from the patient for publication of this case report and accompanying images. A copy of the written consent is available for review by the Editor-in-Chief of this journal on request.

## Provenance and peer review

Not commissioned, externally peer-reviewed.

## Ethical approval

This study was reviewed and approved by the Institutional Review Board and Ethical Committee Dr. Cipto Mangunkusumo, a national reference, and teaching hospital. Patient medical records were maintained under applicable medical ethical standards.

## Funding

This research did not receive any specific grant from funding agencies in the public, commercial, or not-for-profit sectors.

## Guarantor

Gatot Purwoto.

## Research registration number

None declared.

## CRediT authorship contribution statement

Gatot Purwoto: conceptualization, methodology, resources, supervision. Gatot Purwoto, Kelli Julianti, Nathaniel Herlambang, Ilham Utama Surya: writing-original draft preparation, investigation, visualization, writing-review and editing. Gatot Purwoto, Tricia Dewi Anggraeni: supervision, data curation, editing. Gatot Purwoto, Primariadewi Rustamadji, Kelli Julianti: visualization, analyzing, and interpretation data.

## Declaration of competing interest

The authors declare that we have no financial or personal relationship that may have inappropriately influenced us in writing this article.
